# Wearable Carbon Nanotube-Based Biosensors on Gloves for Lactate

**DOI:** 10.3390/s18103398

**Published:** 2018-10-11

**Authors:** Xiaojin Luo, Weihua Shi, Haoming Yu, Zhaoyang Xie, Kunyi Li, Yue Cui

**Affiliations:** 1 College of Engineering, Peking University, Beijing 100871, China; luoxiaojin@pku.edu.cn (X.L.); sswwhh@pku.edu.cn (W.S.); 18210352569@pku.edu.cn (H.Y.); colin0823@pku.edu.cn (K.L.); 2 Department of Electrical Engineering and Computing Systems, University of Cincinnati, Cincinnati, OH 45221, USA; xiezg@mail.uc.edu

**Keywords:** biosensor, wearable, glove, carbon nanotube, amperometric, lactate, enzyme

## Abstract

Developing a simple and direct approach for interfacing a sensor and a target analyte is of great interest for fields such as medical diagnosis, threat detection, food quality control, and environmental monitoring. Gloves provide a unique interface for sensing applications. Here, we show for the first time the development of wearable carbon nanotube (CNT)-based amperometric biosensors painted onto gloves as a new sensing platform, used here for the determination of lactate. Three sensor types were studied, configured as: two CNT electrodes; one CNT electrode, and an Ag/AgCl electrode, and two CNT electrodes and an Ag/AgCl electrode. The sensors are constructed by painting the electrodes using CNT or Ag/AgCl inks. By immobilizing lactate oxidase onto the CNT-based working electrodes, the sensors show sensitive detections of lactate. Comparison of sensor performance shows that a combination of CNT and Ag/AgCl is necessary for highly sensitive detection. We anticipate that these findings could open exciting avenues for fundamental studies of wearable bioelectronics, as well as practical applications in fields such as healthcare and defense.

## 1. Introduction

The integration of bioelectronic sensors into wearable platforms is expected to open up significant opportunities for medical diagnosis, environmental monitoring, and defense applications. A few studies have examined wearable bioelectronic sensors integrated with wearable substrates such as contact lenses [1,2], and bandages [3]. Despite several advances, the field remains nascent, and a wide range of fundamental studies, as well as practical applications, are vital for the further development of wearable bioelectronic sensors based on other wearable substrates [4,5,6,7,8].

Gloves, worn on hands, are disposable wearable materials [9,10]. Integrating bioelectronic sensors onto gloves can provide unique opportunities in scenarios such as healthcare, environmental monitoring, food quality control, and threat detection [6]. Recently, a few glove-based sensors have been developed for the detection of several parameters such as motion [11,12], temperature [13], and virtual motion [14].

Lactate is present in sweat, blood, saliva, and urine, and it is an important biomarker for a variety of clinical analyses [15,16,17,18,19]. Lactate in food can be important for determining food quality [20,21], and lactate in cell cultures can indicate the cell condition and status [22,23]. Many conventional biosensors based on hard substrates have been developed for the detection of lactate [24,25,26], but to provide more versatile platforms, it is desirable to develop a wearable, glove-based biosensor for the detection of lactate. Carbon nanotubes (CNTs) are nanoscale cylinders made of carbon atoms, exhibit extraordinary electrical properties, such as high electron mobility, and have proven to show excellent sensing performance when being used as electrodes in biosensors [8,27,28].

In this work, we demonstrate for the first time the combination of the flexible glove substrate and the excellent electrical properties of CNTs to develop wearable CNT-based glove sensors for the detection of lactate. Furthermore, we cross-compare the performance variance among three types of sensing systems, and we reach a solid conclusion about the testing sensitivity of these sensor structures. An amperometric sensor consists of working, reference, and counter-electrodes. All three electrodes are covered by buffer solution in the measurement. The reference electrode shows a stable and fixed (reference) potential in the buffer solution without any passing current. The working electrode has a potential against the reference electrode, and in the amperometric measurement, it is a constant value. The working electrode and the counter electrode form a circuit where the current is measured. The oxidation or reduction reaction due to the presence of analyte occurs on the working electrode, which further changes the current in the circuit. Thus, from the change of the current signal, the concentration of analyte can be determined. Three electrode configurations are studied here. The first configuration employs two CNT-based electrodes that function as the working and reference/counter electrodes, respectively. The second configuration employs one CNT working electrode and one Ag/AgCl reference/counter electrode. The third type employs two CNT working and counter electrodes, and one reference Ag/AgCl electrode. These sensors are constructed by painting with CNT or Ag/AgCl inks. Lactate oxidase (LOD) is immobilized onto the CNT-based working electrode, which catalyzes lactate to generate H_2_O_2_, and at a potential of 0.6 V for the working electrode versus the reference electrode, H_2_O_2_ is oxidized to generate a signal response.

## 2. Materials and Methods

### 2.1. Apparatus and Chemicals

A potentiostat (Ivium CompactStat) with IviumSoft Electrochemistry software was purchased from Ivium Technologies (Eindhoven, The Netherlands). A spectrophotometer (Agilent CARY5000) was purchased from OSIC Holding Group Co., Ltd. (Beijing, China). A lactate acid assay kit was purchased from Solarbio Science & Technology Co., Ltd. (Beijing, China). Ag/AgCl ink with a 50:50 Ag:AgCl ratio was purchased from Gwent Electronic Materials Ltd. (Pontypool, UK). Carbon nanotubes (optical density 10–20 nm, length 10–30 μm, Ash: <1.5 wt %, specific surface area: >200 m^2^/g, electrical conductivity: >100 s/cm, purity: >95%) were purchased from Xfnano (Nanjing, China). Toluene was purchased from Beijing Tongguang Fine Chemical Company (Beijing, China). Nitrile gloves were purchased from Shijiazhuang Hongrui Inc. (Shijiazhuang, China). Sodium hydroxide was purchased from Beijing Chemical Works (Beijing, China). Sodium dihydrogen phosphate was purchased from Tianjin Fuchen Chemical Reagent Factory (Tianjin, China). Lactate oxidase was purchased from Toyobo Co., Ltd. (Osaka, Japan). Sodium l-lactate, glutaraldehyde, urea, uric acid, and hydrogen peroxide were purchased from Sigma-Aldrich Inc. (Beijing, China). Heating glue was from Xnyhc, Inc. (Beijing, China).

### 2.2. Sensing Electrode Preparation

Carbon nanotubes (CNTs) were dissolved into toluene at a concentration of 0.01 mg mL^−1^. Thin lines of CNTs and Ag/AgCl inks (length 15 mm, width 2 mm) in film status were manually deposited onto the clean glove surface with an auto pipette. For the sensor configuration utilizing two CNTs, two lines of CNTs were manually deposited onto a glove as the working/counter electrode and the reference electrode, respectively, at 4 mm spacings. For the sensor utilizing one CNT electrode as the working/counter electrode and one Ag/AgCl electrode as the reference electrode, each electrode was manually deposited from the respective inks at 4 mm spacings. For the configuration utilizing one CNT working electrode, one CNT counter electrode, and an Ag/AgCl reference electrode, each electrode was manually deposited from the respective ink at 1 mm spacing. After deposition, the ink-painted glove was dried at 60 °C for 20 min. Another thin layer of heating glue (about 2 mm thick) was added on the circle top of the electrode, except at the sensing area, and allowed to dry.

### 2.3. Enzyme Functionalization

Glutaraldehyde solution (2%) and a lactate oxidase solution (LOD, 10 U μL^−1^) were mixed at a 1:1 volume ratio, and 3 μL of the mixture was dropped onto the working electrode to immobilize the enzyme, then allowed to dry overnight in a refrigerator at approximately 4 °C. The next day, the sample was removed from the refrigerator, allowed to equilibrate to room temperature (21 °C), and incubated with phosphate-buffered saline solution (PBS) for approximately 1 h prior to conducting any measurements.

### 2.4. Sensing Measurements

The sensing measurements were performed at room temperature (21 °C) with the measuring device at a potential of 0.6 V at the working electrode versus the reference electrode. To detect a target analyte, 100 μL of PBS (50 mM, pH 7.0) was added to cover the sensing area (2 mm length by 2 mm width). When the signal stabilized, 5 or 7.5 μL of the analyte at different concentrations was then added into the buffer sequentially. The resulting current-verses-time curves were recorded. Current increased upon the injection of an analyte. Another concentration of the analyte was added until the current stabilized. Calibration curves were plotted for current response versus analyte concentration. The cyclic voltammetry measurements of the three types of sensors were performed by sweeping the potential of working electrode versus the reference electrode from −1.0 V to 1.0 V.

To test the sensing performances with deformations, a three-electrode sensing system on a glove substrate was utilized. The glove substrate was deformed from the center of the sensing electrode with folding back (180°) and elongation by 10% up to 50 times. During the deformation, the sensor response to 0.5 mM was recorded.

Human sweat samples were obtained under three conditions: running 1000 m for 4 min, with an average speed of 4.17 m/s; cycling 5000 m for 20 min, with an average speed of 4.17 m/s; and jogging 1700 m for 12 min, with an average speed of 2.36 m/s. Abundant rest was taken after each form of exercise, to make sure that the testing experiment and the subject’s status were approximately the same. Cycling was carried out at noon at a temperature of 31 °C, and after this, the testing subject took a rest for about 1 h before jogging. The testing subject’s heart rate returned to be normal completely before jogging. Running was carried out on the next day at noon at a temperature of 31 °C. For the measurement of lactate concentration in a human sweat sample, a three-electrode sensing system was utilized. A 99 μL volume of PBS (50 mM, pH 7.0) was added to cover the sensing area (2 mm length by 2 mm width). When the signal stabilized, 1 μL of a sweat sample was added into the PBS, and the current response was recorded. Then, the current response was compared with the calibration curve of the sensor to determine the lactate concentration in the sweat sample. To detect the lactate concentration in a sweat sample with a spectrophotometer, the sweat sample was prepared according to the manual of the lactate acid assay kit from Solarbio, and its absorbance was measured under a wavelength of 570 nm. The standard lactate solutions were prepared and measured with a spectrophotometer according to the manual, as well as to plot a calibration curve for lactate. Since the lactate concentration in a sweat sample was in proportion to the absorbance, it could be calculated when being compared with the calibration curve.

## 3. Results and Discussion

**Figure 1:**
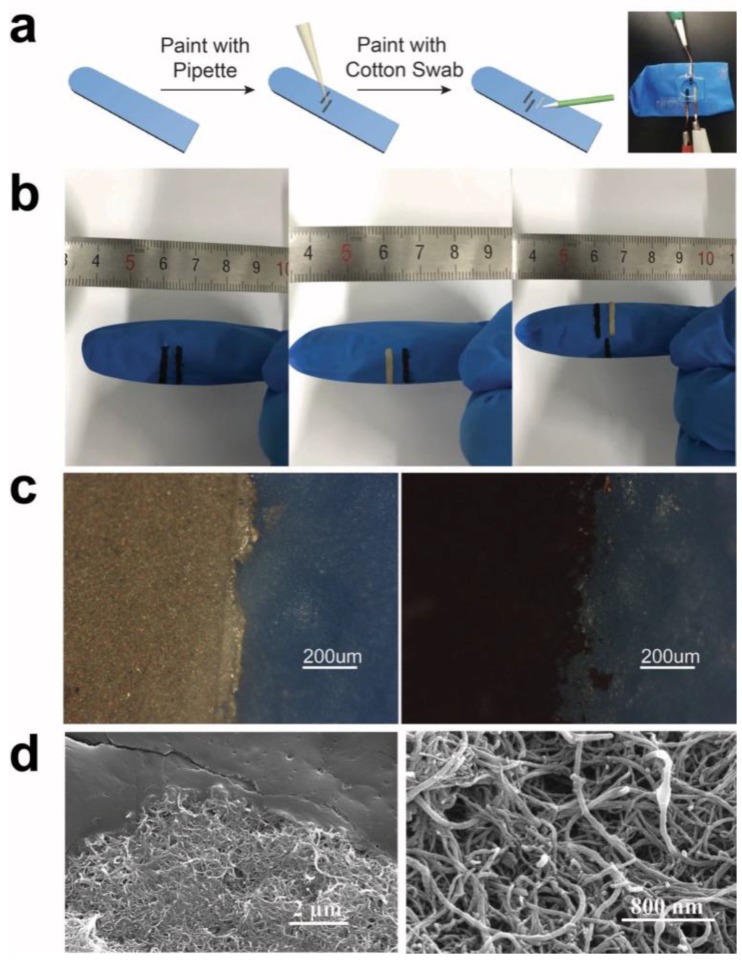
Optical characterization of the glove-based biosensor. (**a**) Schematic illustration of the fabrication process of the sensor and the connection method. From left to right: Blank glove; Depositing CNT solution onto the glove surface with auto pipettes; painting the Ag/AgCl electrode with cotton swabs; connecting electrodes with potentiostat with copper wires. (**b**) Camera images of the CNT-based glove biosensors. **Left**: a sensor based on two CNT electrodes. **Middle**: a sensor based on a CNT and an Ag/AgCl electrode based sensor. **Right**: a sensor based on two CNT electrodes and an Ag/AgCl electrode. (**c**) Optical image of the interface between CNT electrode and the substrate (**left**) and magnification of CNT electrode (**right**). (**d**) SEM images of the CNT electrode. **Left**: Interface between the CNT electrode and the glove substrate. **Right**: the surface of CNT electrode.

Figure 1 shows the schematic illustration of the sensor construction process and the connection method (Figure 1a), the camera images of the sensors (Figure 1b), the optical images of the CNT-electrode, and the Ag/AgCl electrode (Figure 1c), and the SEM images of the CNT electrode (Figure 1d). Figure 1a shows the construction process of a three-electrode system, and the two-electrode systems were done with similar processes. As shown in Figure 1a, the two CNT electrodes were painted from a CNT solution with a pipette as the working and counter electrodes, and after this, an Ag/AgCl electrode was painted from an Ag/AgCl paste with a cotton swab as the reference electrode. After this, the CNT and Ag/AgCl electrodes were connected to leads from the potentiostat with copper wires, as shown in the image in Figure 1a. Figure 1b shows camera images of the three glove-based sensor configurations: two CNT working- and reference/counter electrodes (Figure 1b left); one CNT working electrode and one Ag/AgCl reference/counter electrode (Figure 1b middle); two CNT working- and counter electrodes with one Ag/AgCl reference electrode (Figure 1b right). Figure 1c shows the optical image of the electrodes painted with CNT (Figure 1c left) and Ag/AgCl ink (Figure 1c right), respectively. As shown in the camera images and optical images, the three sensor configurations were successfully constructed on the fingers of the gloves. The CNT and Ag/AgCl electrodes had dimensions of 2 mm, with 4 mm spacings between the electrodes. Figure 1d shows the SEM images of the CNT electrode. Figure 1d left shows the interface between the CNT electrode and the glove substrate, from which it can be seen clearly that strands of CNTs were able to attach to the substrate through Van der Waals force without any glue, and bundles of carbon nanotubes intertwined with each other and stack layer-by-layer to form the whole electrode. Figure 1d right is the magnification of the CNT electrode, which exhibits the intertwining nanotubes, and this intertwining relationship arouses strong Van der Waals force between CNTs and holds the whole electrode together. The average resistance of the CNT electrode was 1.66 kΩ, while the average resistance between the working and counter electrodes with covering buffer was 5.83 MΩ. The average thickness of the CNT layers was measured with a micrometer as 3.3 μm, while the average thickness for the Ag/AgCl electrodes was 4.75 μm. These results demonstrate that these three types of sensors were successfully fabricated onto the wearable glove substrates.

**Figure 2:**
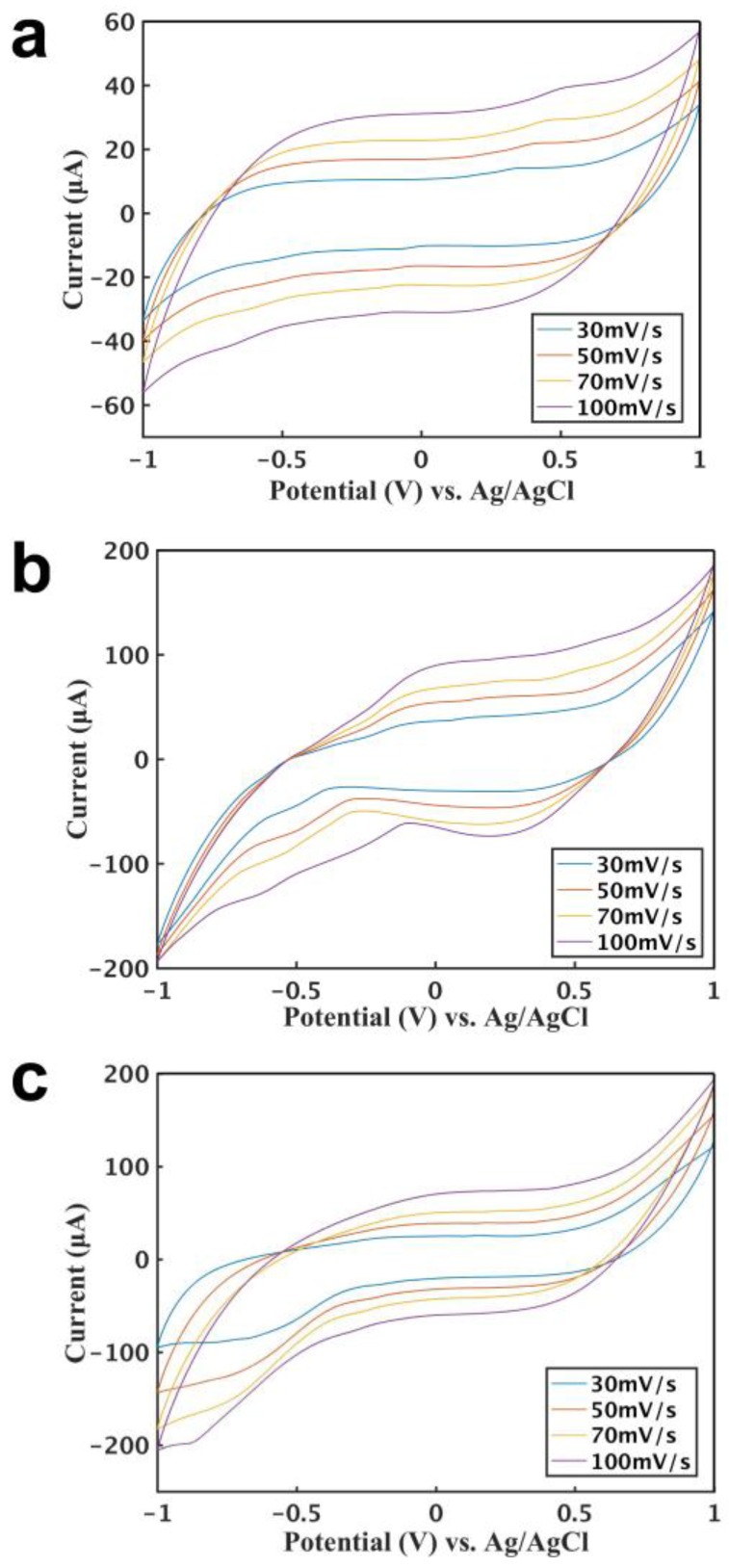
Characterization of the three styles of sensors for the CV curves at different scan rates. (**a**) Cyclic voltammetry curve for 2 mM H_2_O_2_ based on two CNT electrodes. (**b**) Cyclic voltammetry curve for 2 mM H_2_O_2_ by the sensor based on a CNT electrode and an Ag/AgCl electrode. (**c**) Cyclic voltammetry curve for 2 mM H_2_O_2_ by the sensor based on two CNT electrodes and an Ag/AgCl electrode.

Figure 2 shows the cyclic voltammograms of three types of sensors at different scanning rates in a buffer solution containing 2 mM H_2_O_2_. In Figure 2a, the cyclic voltammograms of a CNT-CNT sensor showed that the current increases with the increase of the working electrode, and that H_2_O_2_ was oxidized on the electrode to produce the currents at different potentials. When the scanning rate increased, the current also increased. The sensors showed a strong current response under 0.6 V, indicating that H_2_O_2_ underwent a more intense oxidation reaction at this potential, and 0.6 V was used for further studies. Figure 2b shows the cyclic voltammograms of a CNT-Ag/AgCl sensor, and Figure 2c shows that of a CNT-CNT-Ag/AgCl sensor, and the curves from these two types of sensors exhibit similar performances. The current signal ranges are basically the same, but they are much larger than the first sensor.

**Figure 3:**
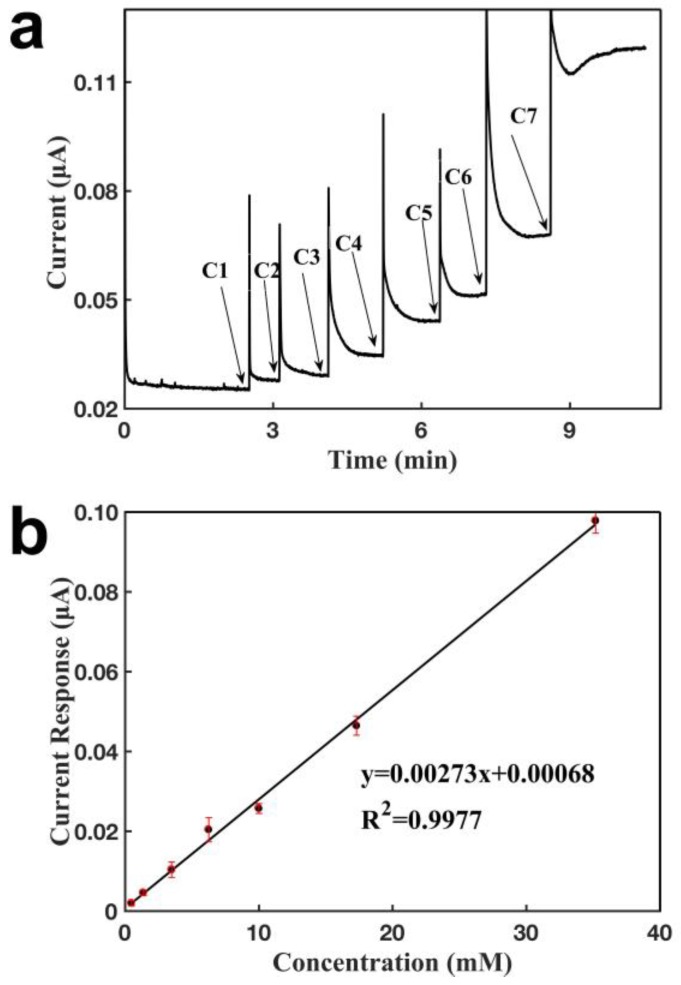
Characterization of the glove sensor based on two CNT electrodes for the detection of H_2_O_2_. Operating potential: 0.6 V of working electrode vs reference electrode (CNT). (**a**) Current-versus-time response curve upon the additions of H_2_O_2_. C1: 0.48 mM, C2: 0.89 mM, C3: 2.11 mM, C4: 2.77 mM, C5: 3.75 mM, C6: 7.30 mM, C7: 17.88 mM. (**b**) Calibration curve for the detection of H_2_O_2_. Each error bar was from three measurements.

Figure 3a shows the current-verses-time response curve of the glove-based biosensor configured with two CNT electrodes for the detection of H_2_O_2_. H_2_O_2_ was oxidized at the working electrode to generate a signal response. It was observed that the current increased when a given concentration of H_2_O_2_ was added over the sensor. The sensor responded within 1 s, and achieved a new steady state after approximately 1 min. These results demonstrate that sensor current response increased when a higher concentration of H_2_O_2_ was added, and that the sensor had a short response time. The analyte entered the buffer droplet and diffused to the surface of the sensing electrode to become oxidized/reduced. This was because a higher concentration of analyte generates a larger signal response, and it took longer for this higher signal to reach stability. Thus, the response time was slower for higher analyte concentrations. As shown in Figure 3a, the response time for C1 0.48 mM was 49.6 s, whereas that for C7 17.88 mM was 116.6 s. Figure 3b shows the calibration curve for the detection of H_2_O_2_ with the sensor. In the calibration curve, the concentration was relative to the initial buffer concentration, which did not have any analyte, and the concentration was calculated by the overall amount of analyte added into the buffer, divided by the overall volume. A linear relationship was obtained between the current response and the H_2_O_2_ concentration, ranging from 476.0 μM to 35.19 mM. In this configuration, as the two CNT-based electrodes functioned as both the working and reference/counter electrodes, the sensor was not able to complete the corresponding redox reaction effectively, resulting in low electronic exchange efficiency and a lower current density. Therefore, the sensor had a calibration curve with a shallow slope (0.00273 μA mM^−1^; R^2^ = 0.9977), and a large detection limit (216.0 µM). The detection limit was calculated by three times the current noise divided by the slope of the calibration curve (3 N/k). The sensor displayed excellent measurement repeatability, with the slopes of seven calibration curves being measured as 2.42, 2.72, 2.80, 2.63, 2.77, 2.65, and 2.77 nA mM^−1^.

**Figure 4:**
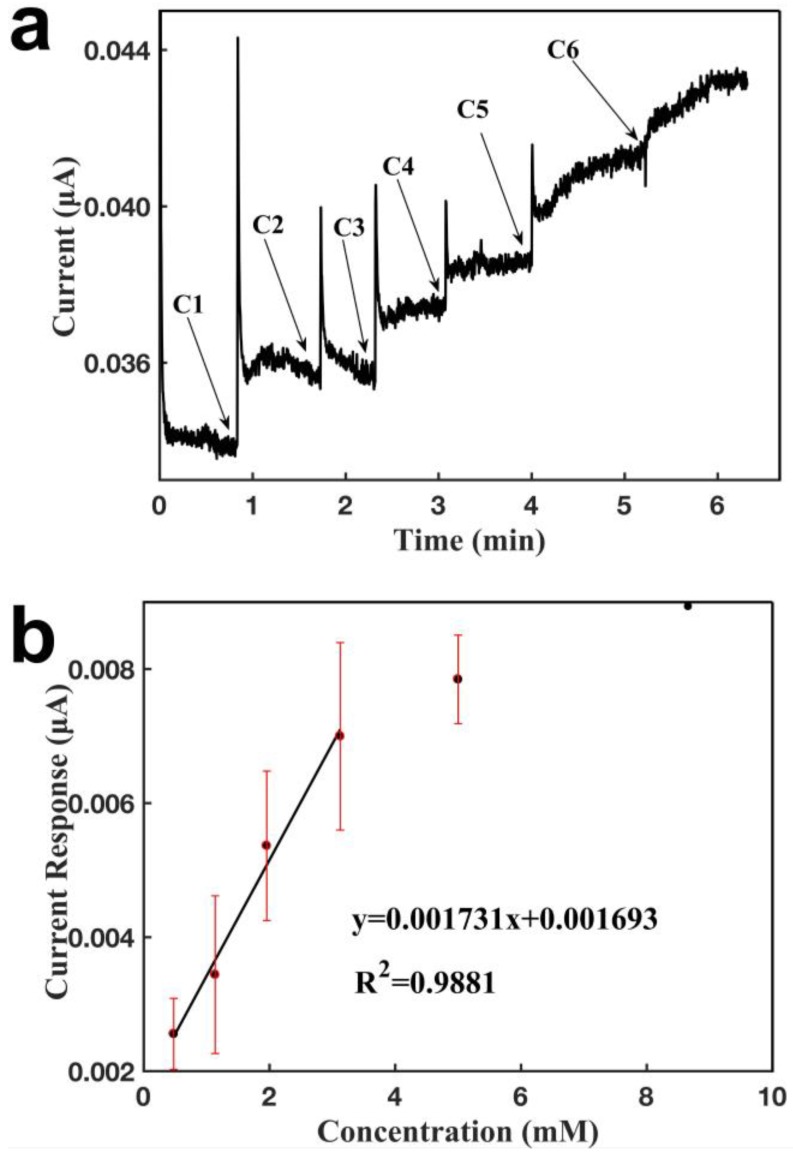
Characterization of a glove sensor based on two CNT electrodes for the detection of lactate. Operating potential: 0.6 V of working electrode vs reference electrode (CNT). (**a**) Current-versus-time response curve upon the additions of lactate. C1: 0.48 mM, C2: 0.66 mM, C3: 0.82 mM, C4: 1.17 mM, C5: 1.88 mM, C6: 3.65 mM. (**b**) Calibration curve for the detection of lactate. Each error bar was from three measurements.

Figure 4 characterizes the performance of the sensor configured with two CNT-based electrodes for detecting lactate. By immobilizing lactate oxidase, lactate is catalyzed to generate H_2_O_2_, which is further oxidized to generate a signal response. As shown in Figure 4a, when a given concentration of lactate was added over the sensor, the sensor responded within 1 s, and the current increased and achieved a new steady state by around 1 min, demonstrating that the sensor had a short response time. The current increased proportionally to lactate concentration within a certain range. Figure 4b shows the biosensor calibration curve for lactate detection, with a linear relationship between the current response and lactate concentration, and a detection range of 476.2 μM to 3.13 mM. The signal saturates at a concentration of 3.13 mM, probably due to the saturation of the analyte for the enzymatic reaction. The sensor showed a detection limit of 258.2 µM and a slope of 0.00173 μA mM^−1^ (R^2^ = 0.9881). Compared with Figure 3, it can be seen that even when using the same type of sensing electrode, the slope of calibration curve for lactate was lower than that for H_2_O_2_. This result indicates that for the same concentration of lactate as the analyte in the buffer solution, through the enzymatic reaction on the working electrode, the generated H_2_O_2_ from lactate on the electrode does not have the same concentration as the lactate concentration in the buffer solution. Due to the involvement of the enzymatic reaction and the difficulty of controlling small current signals, the uncertainty of the response signals increased, which resulted in large error bars for the detection. The signal for lactate was noisier than that for H_2_O_2_, which due to the influence of the enzyme matrix on the working electrode, such as the bioactivity of LOD and the diffusion barrier. The sensor showed excellent repeatability, with slopes of the calibration curves for lactate of 0.00154, 0.00125, 0.00177, 0.00127, 0.00173, and 0.00185 μA mM^−1^.

**Figure 5:**
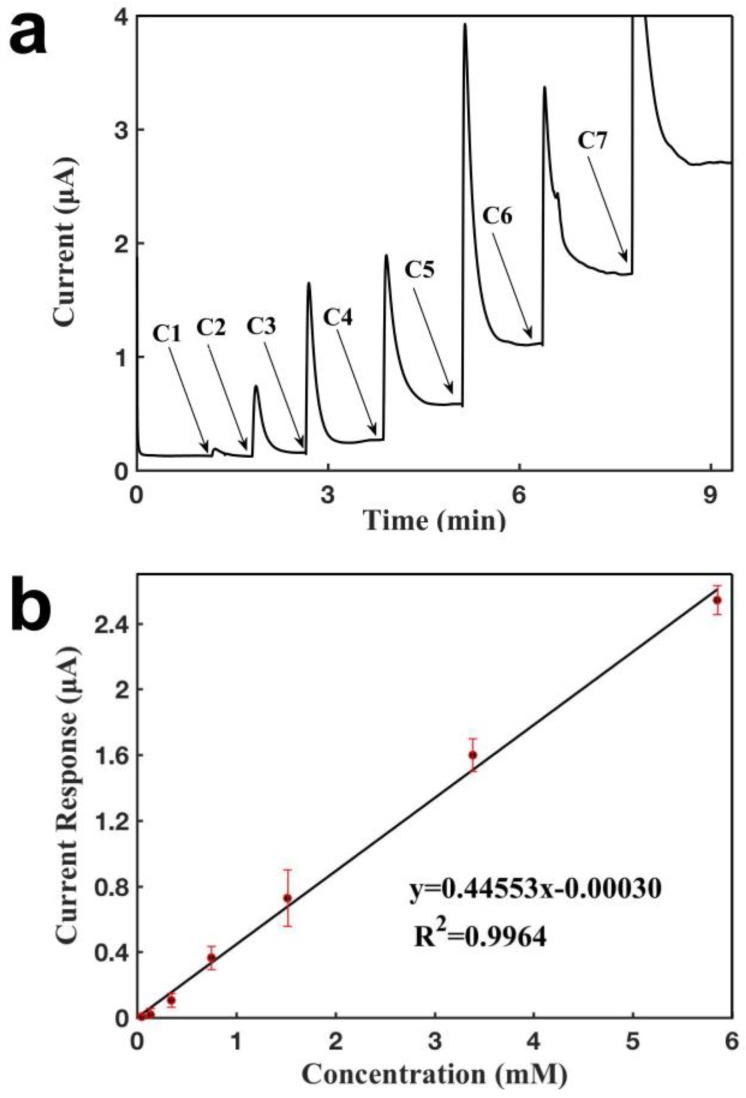
Characterization of a glove sensor based on a CNT electrode, and on an Ag/AgCl electrode for the detection of H_2_O_2_. Operating potential: 0.6 V of the working electrode vs reference electrode (Ag/AgCl 50:50). (**a**) Current-versus-time response curve upon the additions of H_2_O_2_. C1: 0.048 mM, C2: 0.089 mM, C3: 0.30 mM, C4: 0.40 mM, C5: 0.77 mM, C6: 1.86 mM, C7: 2.47 mM. (**b**) Calibration curve for the detection of H_2_O_2_. Each error bar was from three measurements.

Figure 5 characterizes the sensor configured with a CNT electrode and an Ag/AgCl electrode for detecting H_2_O_2_. As shown in Figure 5a, it was observed that the current increased when a given concentration of H_2_O_2_ was added over the sensor, the sensor responded within 1 s, and it achieved a new steady state at around 1 min, which shows that the sensor had a short response time. The current response increased when a higher concentration of H_2_O_2_ was added, with a current increase that was proportional to lactate concentration. Figure 5b shows the calibration curve for H_2_O_2_ detection, showing a linear relationship between the current response and the H_2_O_2_ concentration from 47.6 μM to 5.85 mM. The sensor showed a detection limit of 2.9 µM and a slope of 0.446 μA mM^−1^ (R^2^ = 0.9964). This sensor configuration showed higher sensitivity than that achieved using CNTs as the reference/counter electrode, owing to that Ag/AgCl has a stable potential in the buffer solution whereas CNT does not. Since the potential of the working electrode was versus the reference electrode, the working electrode would be unstable with the CNT electrode as the reference electrode, and it could be lower than that using the Ag/AgCl electrode as the reference electrode. Thus, the sensor with an Ag/AgCl reference electrode had a higher oxidation rate for H_2_O_2_, resulting in a higher sensing signal response and a better detection limit. The sensor shows an excellent repeatability, with slopes for the four calibration curves of 0.429, 0.446, 0.445, and 0.445 μA mM^−1^.

**Figure 6:**
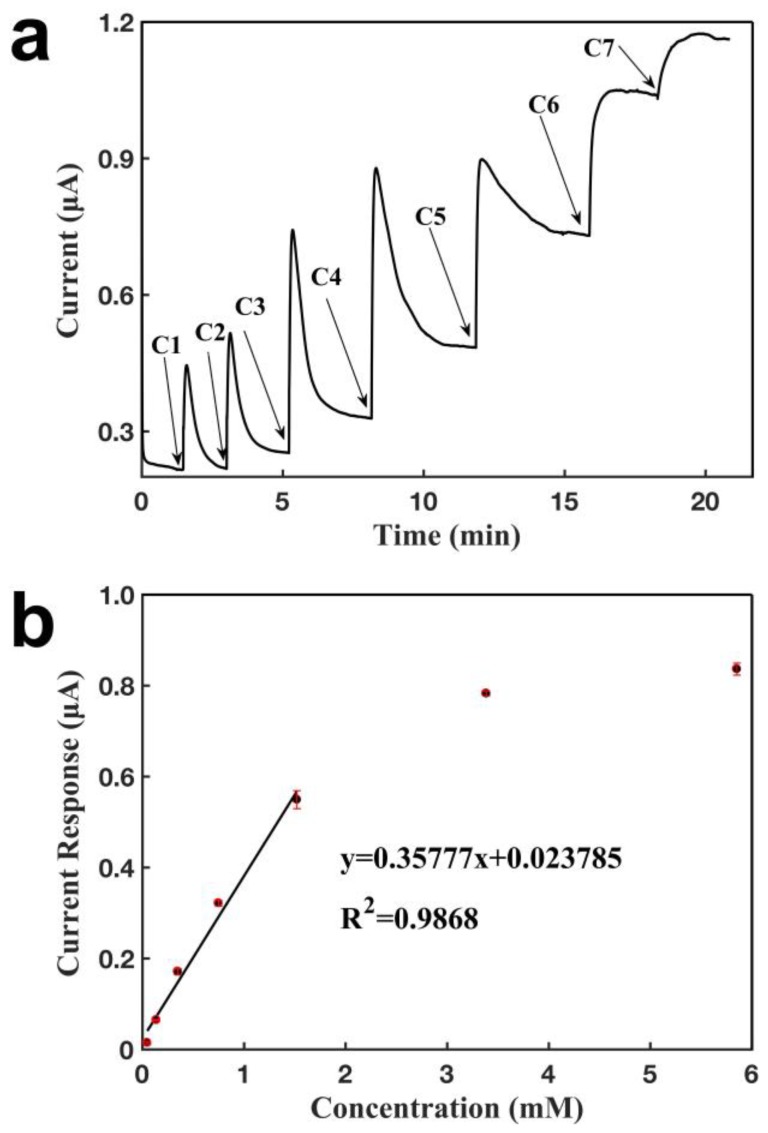
Characterization of glove sensor based on a CNT electrode and an Ag/AgCl electrode for the detection of lactate. Operating potential: 0.6 V of the working electrode vs reference electrode (Ag/AgCl 50:50). (**a**) Current-versus-time response curve upon the additions of lactate. C1: 0.048 mM, C2: 0.089 mM, C3: 0.30 mM, C4: 0.40 mM, C5: 0.77 mM, C6: 1.86 mM, C7: 2.47 mM. (**b**) Calibration curve for the detection of lactate. Each error bar was from three measurements.

Figure 6 characterizes the sensor configured with a CNT electrode and an Ag/AgCl electrode for detecting lactate by the immobilization of lactate oxidase. Similarly, as shown in Figure 6a, it was observed that the sensor responded within 1 s, and the current increased when lactate was added over the sensor, with a new steady state being achieved at around 3 min, showing that the sensor had a short response time for a given concentration. The smaller sensing signal of CNT-CNT sensor contributed to a faster response time compared with that of CNT-Ag/AgCl sensors upon an addition of a lactate concentration, since the sudden uplift of current is smaller, and a balance and a stability of the current should also be regained faster. The current response increased in proportion to higher lactate concentration within a certain range. Figure 6b shows the calibration curve for lactate detection. A linear relationship was obtained between the current response and the lactate concentration, with a detection range of 47.6 μM to 1.52 mM. The sensor had a detection limit of 2.5 µM and a slope of 0.358 μA mM^−1^ (R^2^ = 0.9868). The signal saturated at a concentration of 1.52 mM, probably due to the saturation of the analyte for the enzymatic reaction. The sensor shows excellent measurement repeatability and a small standard deviation, with calibration curves for five measurements having slopes of 0.342, 0.325, 0.389, 0.353, and 0.330 μA mM^−1^.

**Figure 7:**
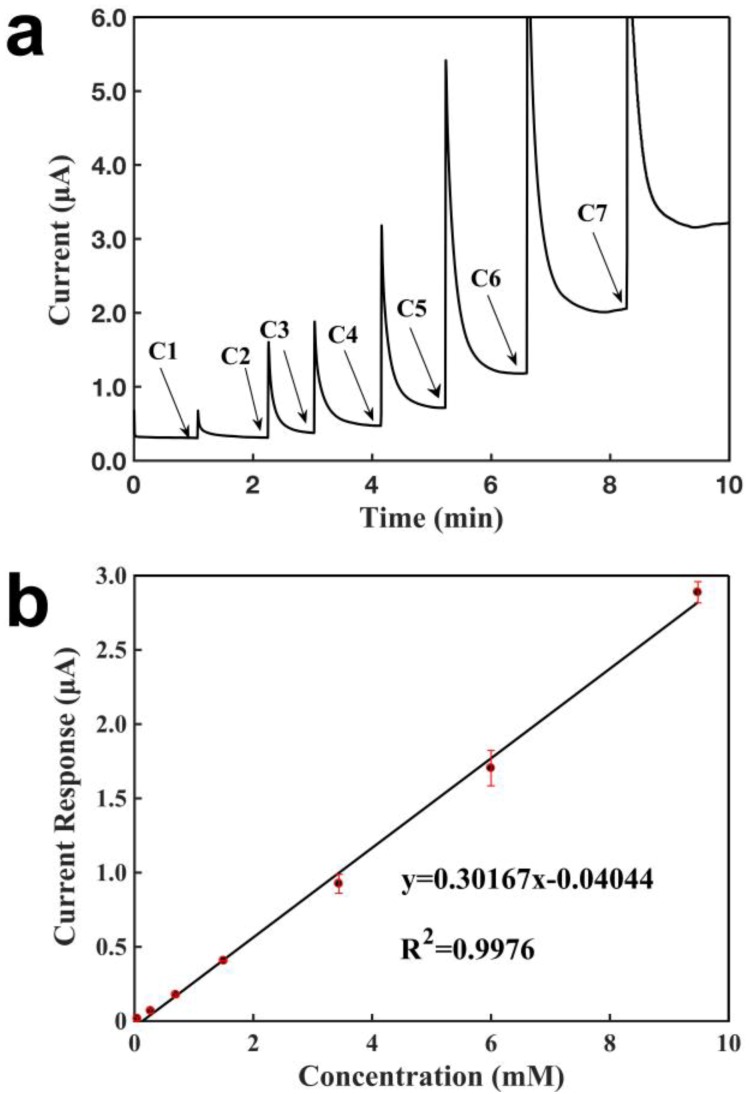
Characterization of a glove sensor based on two CNT electrodes and an Ag/AgCl electrode for the detection of H_2_O_2_. Operating potential: 0.6 V of working electrode vs reference electrode (Ag/AgCl 50:50). (**a**) Current-verses-time response curve upon the additions of H_2_O_2_. C1: 0.048 mM, C2: 0.089 mM, C3: 0.30 mM, C4: 0.40 mM, C5: 0.77 mM, C6: 1.86 mM, C7: 2.47 mM. (**b**) Calibration curve for the detection of H_2_O_2_. Each error bar was from three measurements.

Figure 7 characterizes the three-electrode configuration, with CNT for the working and counter electrodes, and the Ag/AgCl reference electrode, for detecting H_2_O_2_. As shown in Figure 7a, the current increased when a concentration of H_2_O_2_ was added over the sensor, the sensor responded within 1 s, and achieved a new steady state in around 1 min, demonstrating a short response time for a given concentration. The current response increased when a higher concentration of H_2_O_2_ was added, and the current increase was proportional to the H_2_O_2_ concentration. Figure 7b shows the calibration curve for the detection of H_2_O_2_ with the glove-based amperometric biosensor. A linear relationship was obtained between the current response and the H_2_O_2_ concentration, ranging from 47.6 μM to 9.48 mM. The sensor showed a detection limit of 1.4 µM, and a slope of 0.302 μA mM^−1^ (R^2^ = 0.9976). The sensors showed an excellent repeatability, with calibration curves of slopes 0.307, 0.292, and 0.298 μA mM^−1^. The Ag/AgCl electrode functioned as both the reference electrode and the counter electrode in the two-electrode sensing system, and consequently, there was a current flowing in the Ag/AgCl electrode to be polarized, resulting in a higher electrode potential than that in the three-electrode sensor. This further increases the absolute potential of the working electrode, and generated a higher rate of H_2_O_2_ oxidation, so that the calibration slope for this three-electrode sensor was lower than that for the two-electrode sensor with CNT and Ag/AgCl as the electrode.

**Figure 8:**
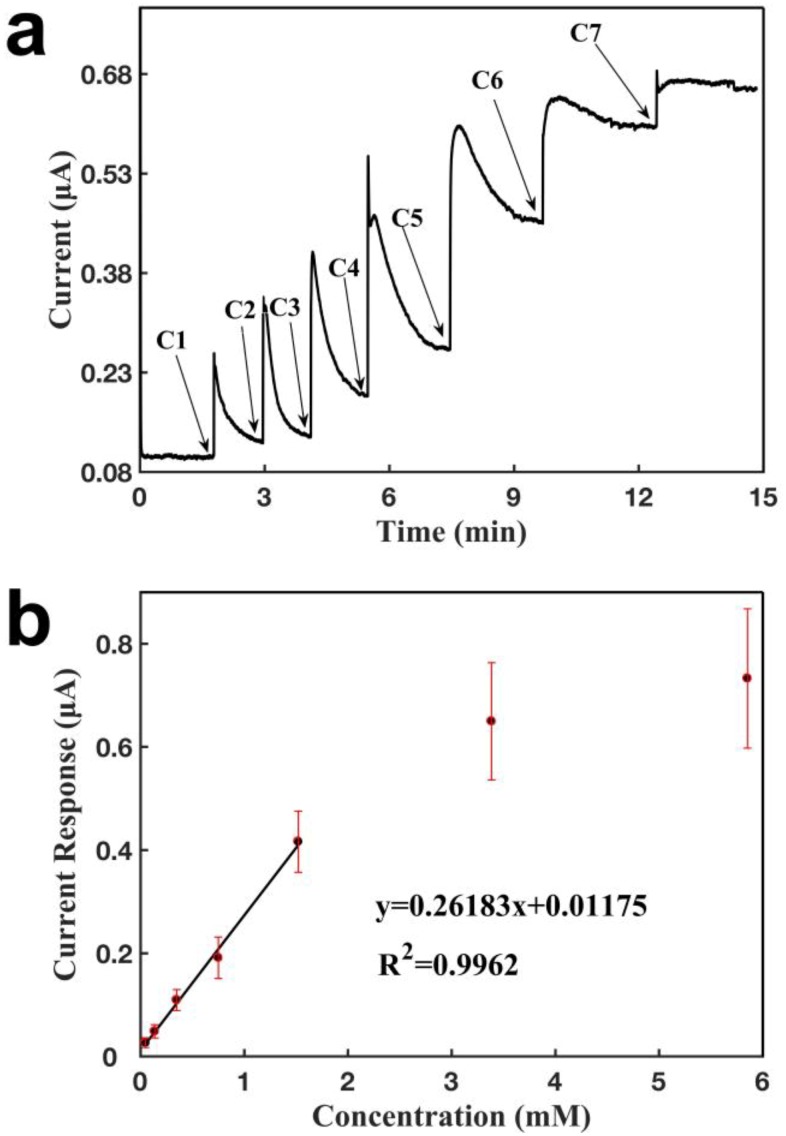
Characterization of a glove sensor based on two CNT electrodes, and an Ag/AgCl electrode for the detection of lactate. Operating potential: 0.6 V of the working electrode vs reference electrode (Ag/AgCl 50:50). (**a**) Current-verses-time response curve upon the additions of lactate. C1: 0.048 mM, C2: 0.089 mM, C3: 0.30 mM, C4: 0.40 mM, C5: 0.77 mM, C6: 1.86 mM, C7: 2.47 mM. (**b**) Calibration curve for the detection of lactate. Each error bar was from three measurements.

Figure 8 characterizes the three-electrode sensor for detecting lactate via the immobilization of lactate oxidase. Similarly, it was observed that the current increased when lactate was added over the sensor (Figure 8a). The sensor responded within 1 s, and achieved a new steady state by around 3 min, demonstrating that the sensor had a short response time for a given concentration. The current response increased when a higher concentration of lactate was added, and the current increase was proportional to the lactate concentration within a certain range. The calibration curve for lactate detection (see Figure 8b) showed a linear relationship between the current response and the lactate concentration, with a detection range of 47.6 μM to 1.52 mM. The sensor showed a detection limit of 6.0 µM and a slope of 0.262 μA mM^−1^ (R^2^ = 0.9962). The signal saturated at a concentration of 1.52 mM, due to the saturation of the analyte for the enzymatic reaction. The results showed good repeatability, with slopes of 0.272, 0.286, 0.226, and 0.237 μA mM^−1^.

The results demonstrate that all three of the sensor configurations can be successfully painted onto glove substrates, and can be used to detect H_2_O_2_ and lactate. The sensors show a fast response time and highly sensitive detection. Furthermore, via the immobilization of other enzymes or bioreceptors, this platform provides new possibilities for constructing other biosensors on gloves in order to detect a variety of other analytes in healthcare, environmental monitoring, and defense applications.

The performance of the three CNT electrode-based glove sensor was compared and analyzed. All three configurations showed detection ranges from micromolar to millimolar concentrations for H_2_O_2_ and lactate. The high sensitivity of the sensor with Ag/AgCl as the reference electrode is critical. Both the two-electrode and three-electrode configurations with Ag/AgCl as the reference electrode provided highly sensitive detection, with similar slope values. The two-electrode CNT system with the CNT reference electrode had a much shallower slope, indicating that this sensor was less sensitive than the two configurations that utilized the Ag/AgCl reference electrodes, and that CNT was less effective as a reference electrode.

The sensor could be worn inside or outside the glove. By applying the sensor outside the glove, it can be used for a variety of applications, such as enabling clinicians to analyze body fluid samples, environmental researchers to detect pollutants, or food researchers to determine food quality. By applying the sensor inside the glove, it can be used for real-time monitoring of the wearer’s health status.

**Figure 9:**
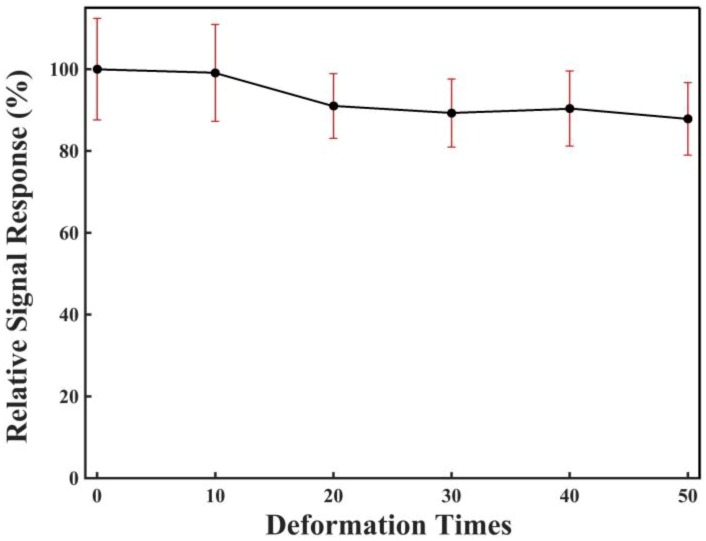
Deformation of the glove sensor based on a three-electrode sensing system for the detection of lactate. The deformation condition: folding back (180°) and elongation by 10%. Vertical ordinates: The relative signal response was obtained by normalizing the sensing responses after a certain number of times of deformation to the sensing response before deformation. Lactate concentration: 0.5 mM. Three sensors were used for the measurement.

Figure 9 shows the sensor’s stability against physical deformation based on the three-electrode sensing system. As shown in the figure, it can be seen that these sensors showed little deviance in performance after a high intensity of physical deformation. The sensors could can show almost 90% of the initial sensing response, even after 50 cycles of harsh physical deformation. Here, all the deformation was within the plastic deformation range of the glove, and the electroanalysis was conducted only after the deformation was restored; therefore, there was no addition restraint imposed on or within the electrode during the signal test, which differentiated it from the pressure and tactile sensors [29,30]. Furthermore, the mechanism behind the sensing signal here was a bit different from the pressure sensors: the signal here came from the electrochemical reactions on the electrodes because of H_2_O_2_, which was produced from the LOD-catalyzed reaction. More specifically, the signal came from the oxidation reaction on the working electrode, the reduction reaction on the counter electrode, and the electron transfer on the electrodes [31]. Therefore, the resistance or capacitance change of the electrodes has less effect on the sensing signals. As bending and stretching are the predominant deformations in the actual use, the results indicate that the glove sensor has a high stability against physical deformation for real applications. However, although the glove sensor showed a relatively strong resistance against deformation, the sensing performance could still decrease after dozens of stretching and folding events. Therefore, to assure the accuracy, we believe that glove sensors should preferably be viewed as for single use.

Before testing real human sweat samples, we tested ascorbic acid, glucose, uric acid, and urea as the disturbances. Glucose and urea showed no current response after being added onto the sensor. Therefore, although urea is rich in human perspiration [32], it poses no interference to the calculation of the lactate concentration. Ascorbic acid and uric acid showed a clear current response, and the calibration curve for ascorbic acid had a slope of 0.309 μA·mM^−1^, and uric acid had a slope of 0.555 μA·mM^−1^, at the same magnitude of lactate (tested with the three electrode system). However, the main organic components of sweat do not consist of ascorbic acid [32], which is metabolized through the kidney and disposed in the urine. Although uric acid does exist in human sweat, it concentration was only around 20 μM [33], about a thousandth of the lactate concentration, which makes it too subtle to be taken into calculation as a disturbance. Therefore, we believe that in real application scenarios, there is no such reducing substance that would act as a significant disturbance.

**Figure 10:**
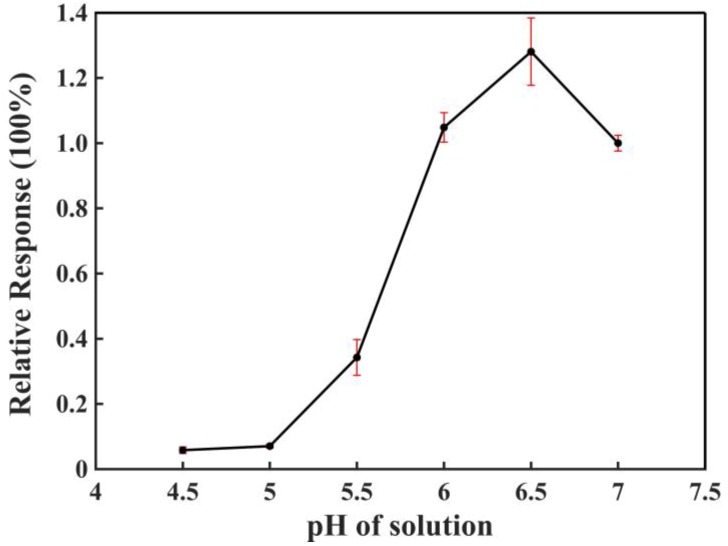
Effect of pH on the detection of lactate based on a three-electrode sensing system. Vertical ordinates: The relative signal response was obtained by normalizing the sensing responses under different pH conditions to the sensing performance under pH 7.0. Lactate concentration: 0.1 mM. Three sensors were used for the measurement.

Figure 10 shows the sensors’ performance under various pH conditions. Human sweat is reported to fall within the pH range from 4.5–7.0, depending on from which part of the body is the sweat collected [34,35]. Sweat samples collected from the lower back ranged from 4.5–6.0, samples from the wrist ranged from 5.0–5.8, samples from the neck ranged from 5.8–7.0, and samples from the chest just below the neck ranged from 6.1–6.7 [34,35]. We prepared PBS buffer solutions ranging from 4.5–7.0 to simulate the real change in sweat pHs, and we found that sensor performance remained at a high level between pH 6.0–7.0, as shown in Figure 10. Although the sensing performance dropped significantly at a pH below 5.5, this was not a fatal problem since we can limit the use of the sensors to within neck or upper chest areas, where the sweat pH is more moderate, to avoid such risks. In this case, we are convinced that our sensor is capable of measuring real human sweat on-site, as long as the sensor is properly used.

**Figure 11:**
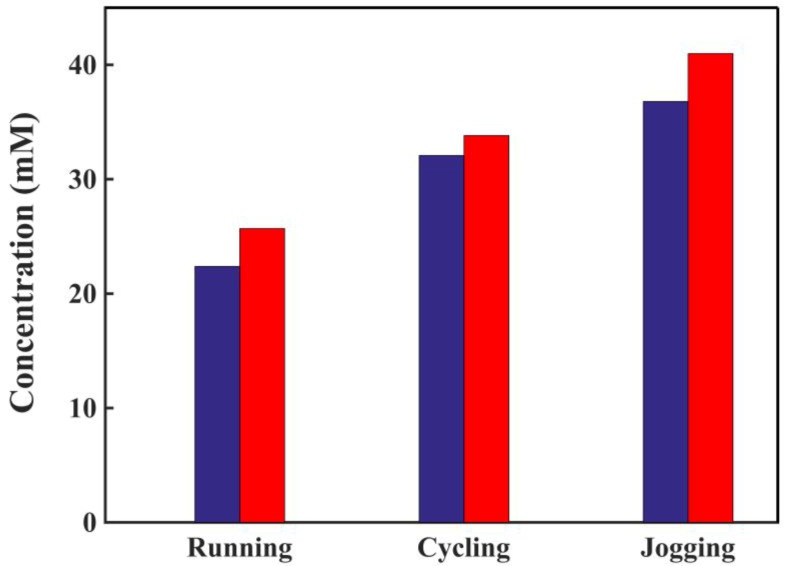
Blue bars: Glove sensor based on two CNT electrodes and the testing results of an Ag/AgCl electrode against real human sweat samples collected after different intensities of exercise. Red bars: Testing results of the same samples with spectrophotometers. Running: 1000 m for 4 min. Cycling: 5000 m for 20 min. Jogging: 1700 m for 12 min.

Figure 11 shows the tests of the sensor for the detection of lactate in real human sweat samples. Three sweat samples were collected from the same subject’s face respectively: running 1000 m for 4 min, with an average speed of 4.17 m/s; cycling 5000 m for 20 min, with an average speed of 4.17 m/s; and jogging 1700 m for 12 min, with an average speed of 2.36 m/s. By using a three-electrode sensor, the human sweat sample generated a signal response, and through the calibration curve, the lactate concentration in the sweat sample was determined, as shown in Figure 11 blue. The running generated a lower concentration of lactate compared with cycling and jogging. The results were consistent with the literature, and a higher intensity of exercise leads to a higher sweat rate, which brings down the lactate concentration, although the overall lactate exertion rate ascends with the exercise intensity [36]. The sensor results were compared with that from a spectrophotometer (Figure 11 red). For running, the sensor showed a result of 22.38 mM, while the spectrophotometer showed 25.69 mM; for cycling, the sensor showed a result of 32.08 mM, while the spectrophotometer showed 33.82 mM; for jogging, our sensors showed a result of 36.81 mM, while the spectrophotometer showed 40.99 mM. These results demonstrated an excellent correlation between the sensor’s measurements and the spectrophotometer’s measurements. The sweat sample collected after all three types of exercises showed a clear discrepancy, which indicates that our sensors are capable of testing actual sweat samples.

## 4. Conclusions

This work demonstrates for the first time the development of wearable CNT-based amperometric biosensors on a wearable glove substrate as a new sensing platform for the detection of lactate. The sensors were constructed by painting CNT and/or Ag/AgCl inks onto gloves, and they function via the immobilization of lactate oxidase onto the sensing electrode. Three sensor configurations were studied and compared, and all of them showed sensitive and rapid detection of lactate. The two-electrode system (with a CNT working electrode and an Ag/AgCl reference/counter electrode) and the three-electrode system (with CNT working- and counter electrodes with Ag/AgCl reference electrode) have higher sensitivities than the two-electrode system employing CNTs as both the working and reference/counter electrodes. CNTs perform excellently as either a working or counter electrode, but less well as a reference electrode. This work also shows that CNT materials can be applied successfully to flexible substrates (in this case glove fingers) as a result of their excellent physical ductility and electrochemical performance. Under a harsh deformation, the sensor shows an excellent stability. The sensors also show the sensitive detection of lactate concentrations in real human sweat samples, and they can differentiate the lactate concentrations under different exercise conditions, which promises practical applications in daily life. The approach described here may facilitate the construction of a variety of other biosensors on gloves, for applications such as medical diagnosis, sports medicine, defense, and environmental monitoring.
